# Increase in rear-end collision risk by acute stress-induced fatigue in on-road truck driving

**DOI:** 10.1371/journal.pone.0258892

**Published:** 2021-10-21

**Authors:** Shunsuke Minusa, Kei Mizuno, Daichi Ojiro, Takeshi Tanaka, Hiroyuki Kuriyama, Emi Yamano, Hirohiko Kuratsune, Yasuyoshi Watanabe

**Affiliations:** 1 Research & Development Group, Hitachi, Ltd., Tokyo, Japan; 2 Laboratory for Pathophysiological and Health Science, RIKEN Center for Biosystems Dynamics Research, Kobe, Hyogo, Japan; 3 RIKEN Compass to Healthy Life Research Complex Program, Kobe, Hyogo, Japan; 4 Osaka City University Center for Health Science Innovation, Osaka, Japan; 5 Department of Medical Science on Fatigue, Osaka City University Graduate School of Medicine, Osaka, Japan; 6 Department of Metabolism, Endocrinology, and Molecular Medicine, Osaka City University Graduate School of Medicine, Osaka, Japan; 7 FMCC Co. Ltd., Osaka, Japan; 8 Division of Health Science, Osaka University Graduate School of Medicine, Osaka, Japan; Universitat de Valencia, SPAIN

## Abstract

Increasing road crashes related to occupational drivers’ deteriorating health has become a social problem. To prevent road crashes, warnings and predictions of increased crash risk based on drivers’ conditions are important. However, in on-road driving, the relationship between drivers’ physiological condition and crash risk remains unclear due to difficulties in the simultaneous measurement of both. This study aimed to elucidate the relationship between drivers’ physiological condition assessed by autonomic nerve function (ANF) and an indicator of rear-end collision risk in on-road driving. Data from 20 male truck drivers (*mean* ± SD, 49.0±8.2 years; range, 35–63 years) were analyzed. Over a period of approximately three months, drivers’ working behavior data, such as automotive sensor data, and their ANF data were collected during their working shift. Using the gradient boosting decision tree method, a rear-end collision risk index was developed based on the working behavior data, which enabled continuous risk quantification. Using the developed risk index and drivers’ ANF data, effects of their physiological condition on risk were analyzed employing a logistic quantile regression method, which provides wider information on the effects of the explanatory variables, after hierarchical model selection. Our results revealed that in on-road driving, activation of sympathetic nerve activity and inhibition of parasympathetic nerve activity increased each quantile of the rear-end collision risk index. The findings suggest that acute stress-induced drivers’ fatigue increases rear-end collision risk. Hence, in on-road driving, drivers’ physiological condition monitoring and ANF-based stress warning and relief system can contribute to promoting the prevention of rear-end truck collisions.

## Introduction

In recent years, traffic crashes due to drivers’ deteriorating health have become a problem worldwide [1]. Even in Japan, traffic crashes caused by drivers’ deteriorating health are increasing, particularly among occupational drivers [2–4]. Health deterioration such as fatigue degrades cognitive and motor performance (e.g., reaction time decline), which can increase the potential risk of traffic crashes [5–7]. To counter this problem, various measures, based on the assessment of drivers’ physiological conditions before and after work, are being undertaken. For example, the Japan Trucking Association is promoting measures to check drivers’ health before they begin their shift, as a means to prevent crashes among truck drivers [8, 9]. Moreover, our research group has also reported that evaluating drivers’ fatigue level, an indicator of physiological condition, before and after work, is effective in estimating the risk of rear-end collision by truck drivers [10].

While traffic crash counter measures based on pre- and post-shift measurement of physiological conditions have been undertaken, investigation into the practical application of efforts utilizing physiological conditions during work to reduce traffic crashes has made limited progress. Given that changes in physiological conditions occur over time [11], utilizing physiological conditions specifically during driving, which accounts for the majority of the shift, may be an important and effective means of reducing traffic crash risk. For example, to prevent drowsy driving, a type of traffic crash risk, a technology that quantitatively evaluates and detects drowsiness during driving based on the driver’s autonomic nerve function (ANF) index obtained from a heart rate variability analysis has been developed [12]. However, the relationship between traffic crash risk and physiological condition has not yet been adequately elucidated, with the exception of physiological conditions such as drowsiness, which are clearly known to pose a risk of traffic crashes [13]. This is mainly due to two challenges.

The first issue is the inability to obtain crash risk assessment data while a vehicle is actually being driven. Since physiological measurements require attaching measuring equipment to vehicles and drivers, it is not possible to analyze the relationship between traffic crash risk and physiological condition from existing crash history data measured from normal vehicles. Therefore, research has mainly been carried out in experimental environments where measuring equipment is attached to vehicles and drivers and a drive simulator is used to simulate conditions that could pose a crash risk [12, 14, 15]. However, it has been reported that the physiological response generated by actual vehicle operation is not reproduced in the laboratory due to adaptation to the experimental environment [16]. Therefore, it is necessary to analyze data obtained during the operation of actual vehicles. Meanwhile, due to such crashes being rare occurrences, it is difficult to obtain sufficient historical data on crashes, including physiological condition data, during actual vehicle operation within the experiment period [10]. Moreover, since the degree of latent risk in the event of a crash not occurring is not represented in the crash history data, it is not suitable for evaluating the effects of changes in physiological condition on crash risk. As outlined above, a crash risk assessment method that does not depend on the occurrence of crashes during actual vehicle operation has yet to be established and consequently, the relationship between risk and physiological condition has been difficult to analyze.

The second issue is that the relationship between crash risk and physiological conditions during vehicle operation has been limited in terms of assessable effects. In the past, crash analysis has largely been conducted using crash history data, which does not include physiological condition data. For example, factor analysis of injury severity due to rear-end collision using random parameters bivariate ordered probit model [17] and severity analysis based on spatio-temporal structure of crash incidence [18] have been previously reported. These studies examined the mean effect of the explanatory variables, explicitly assuming the distribution of the response variables (e.g., Poisson or negative binomial distribution for crash frequency [19]). However, response variables associated with crash risk have been reported to show skewed distribution [19, 20] and it is possible that evaluating only the mean effect of the explanation variable is not adequate for analyzing the relationship. In recent years, quantile regression, which can examine the effect on response variable quantiles without assuming that the response variable has a specific distribution, has been introduced in the field of crash analysis, allowing for more detailed analyses [19–22]. Based on the above, detailed effects analysis, beyond the mean effect, is necessary to analyze the relationship between crash risk and physiological conditions.

The aim of this study was to clarify the relationship between the risk of rear-end collision, which accounts for half of all truck crashes [23], and ANF, evaluated by physiological conditions such as fatigue, during actual vehicle operation [24]. To this end, first, the degree of rear-end collision risk during driving was quantified. Subsequently, the relationship between the estimated rear-end collision risk and the ANF indicator during on-road driving was analyzed. Finally, we conducted a comparative evaluation of ANF during operation of the actual vehicle and pre- and post-work. We found that drivers’ fatigue induced by acute stress increases the risk of rear-end collision.

## Materials and methods

### Participants

Twenty-six male truck drivers (*mean* ± *SD*, = 48.3±8.2 years; range, 28–63 years) without heart disease were recruited from a logistics company to participate in this study. The study protocol was approved by the Institutional Review Boards of RIKEN, Kobe2 (2018–03(4)) and Kansai University of Welfare Sciences (19–02), and the internal review board of Research & Development Group, Hitachi, Ltd., and was conducted in accordance with the Declaration of Helsinki. All participants provided written informed consent prior to enrollment in this study.

### Study design and procedures

All participants’ ANF and working behaviors were monitored over a period of approximately three months. Four participants were excluded from the analysis, as spike-shape outliers of R-R interval (RRI) were chronically observed in their data even while driving, when they hardly moved. Additionally, two more participants were excluded from the analysis because the amount of their data during driving was much smaller compared to the regular driving time of the truck drivers in the company. Accordingly, the data of 20 eligible male participants (*mean* ± *SD*, 49.0±8.2 years; range, 35–63 years) were finally analyzed.

**Fig 1:**
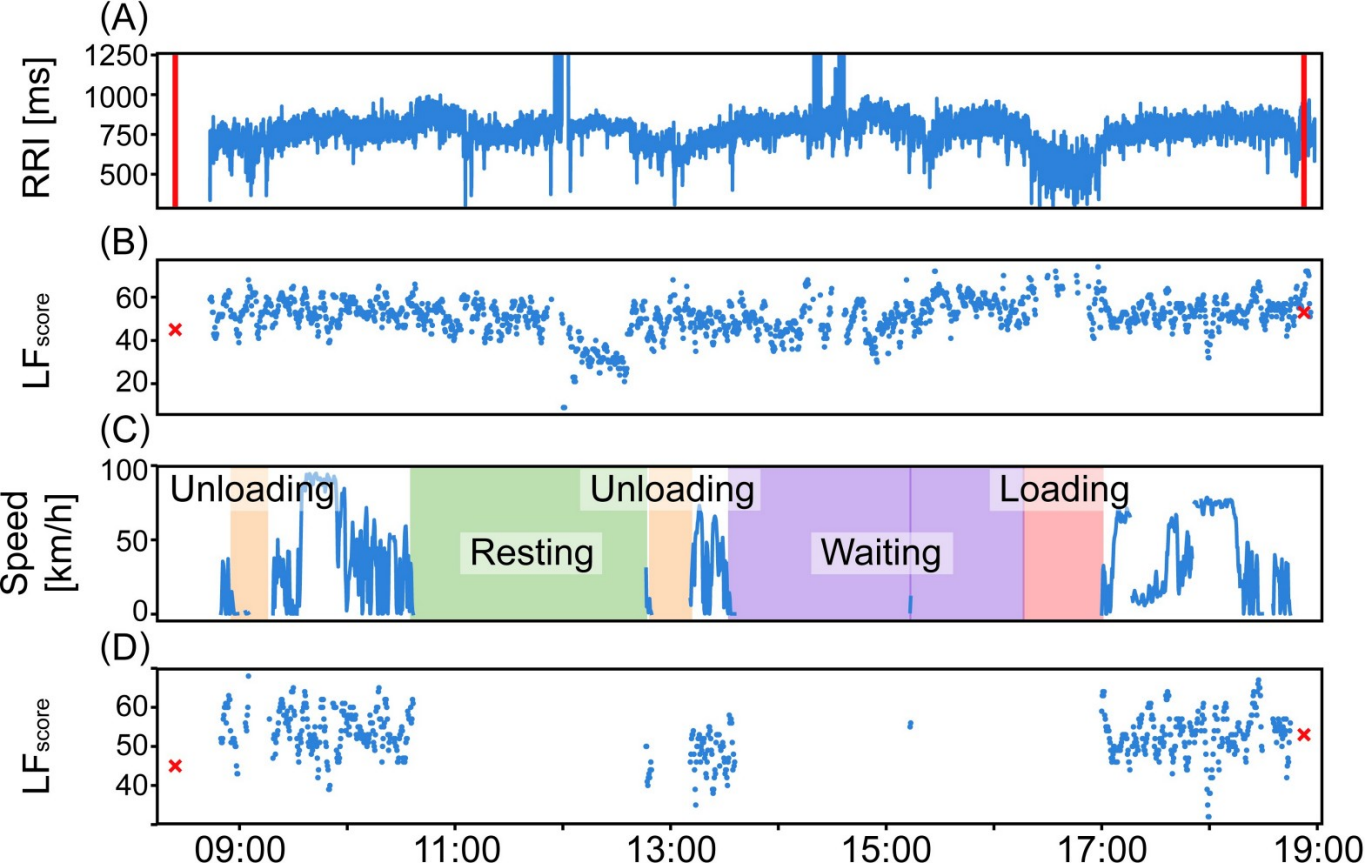
Example of daily obtained data. (A) Obtained RRI time-series in mid-shift. Red perpendicular lines show pre- and post-shift measures. (B) Time series of LF_score_ in mid-shift. Red crosses show LF_score_ in pre- and post-shift. (C) Working behavior including vehicle speed and detailed work shift in mid-shift. (D) Time series of LF_score_ only during driving.

ANF was measured in the mid-, pre-, and post-shift conditions. In the mid-shift condition, drivers’ RRIs were continuously measured using a wearable heart rate sensor (myBeat WHS-1, UNION TOOL CO., Tokyo, Japan) with 1 kHz sampling [25]. During driving work periods in the mid-shift, drivers’ RRIs were stably measured regardless of their body motion caused by their driving behaviors; however, RRI detection sometimes failed in the mid-shift condition except for driving work periods such as loading and unloading work periods (Fig 1). The obtained RRI time-series were divided into windows of 120 s each, and ANF was calculated for each window. In the pre- and post-shift conditions, RRI and ANF were obtained using the fatigue stress measurement system (VM500, Fatigue Science Laboratory, Osaka, Japan), which simultaneously measures electrocardiogram and photoplethysmogram, in the resting eye-closing state for 90 s with 600 Hz sampling [10]. Under all shift conditions, any windows in which misdetection or abnormal intervals exceeded 10% of the total beats, or the calculated heart rate showed outliers, were excluded from ANF calculation due to the instability of the measurement and the low reliability of calculated ANF.

In ANF calculation, various RRI features, such as frequency-domain features were first obtained as the ANF indicators using a similar method as that described in our previous study [10]. Briefly, frequency-domain features were obtained by integrating some frequency bands in the power spectral density (PSD) based on the maximum entropy method, which is adequate for estimating PSD from short-duration RRI [26]. In this study, the low-frequency (LF) components of the 0.04–0.15 Hz band, which mainly represent the degree of activity of the sympathetic nervous system (SNS), and the high-frequency (HF) components of the 0.15–0.40 Hz band, which represent the degree of activity of the parasympathetic nervous system (PNS), were calculated. In addition, the LF/HF ratio, which represents the balance between sympathetic and parasympathetic nerve activity, was also calculated. LF and HF are affected by heart rate and aging [27, 28], and as we cannot control drivers’ heart rates and ages in actual working shifts, we utilized the devised score of LF and HF using a method described in previous studies [10, 29, 30]. The LF deviation score LF_score_ was defined by the following formula:

$${LF}_{score}(LF,age)=\frac{log(\sqrt{LF}/{RRI}_{average})-\mu_{LF}(age)}{\sigma_{LF}(age)/10}+50$$

where, *μ*_LF_(*age*) and *σ*_LF_(*age*) are the mean and standard deviation of *age*, respectively, in the LF standard distribution *N*_LF;*age*_ that approximates a normal distribution. *RRI*_average_ is the average RRI, and log (*z*) is the natural logarithm of *z*. Moreover, the HF deviation score HF_score_ was defined as follows:

$${HF}_{score}(HF,age)=\frac{\sqrt{HF}/{RRI}_{average}-\mu_{HF}(age)}{\sigma_{HF}(age)/10}+50$$

where, *μ*_HF_(*age*) and *σ*_HF_(*age*) are the mean and standard deviation of *age*, respectively, in the HF standard distribution *N*_HF;*age*_ that approximates a normal distribution.

In addition to frequency-domain RRI features, other types of RRI features were also obtained to exploratorily analyze the relationship between collision risk and ANF. As a popular measure of heart rate, average heart rate (AVGHR) was calculated. From the popular time-domain measures, the standard deviation of normal-to-normal R-R intervals (SDNN), NN intervals greater than 50 ms (NN50), and root mean square successive difference (RMSSD) were also calculated, where SDNN mainly reflects SNS activity similar to LF, and NN50 and RMSSD reflect PNS activity similar to HF [31, 32]. Due to the limitation of the measurement system used in the pre- and post-shift conditions, these time-domain features were only obtained in the mid-shift condition.

### Estimation of rear-end collision risk

A method was developed to estimate collision risk solely from vehicle behavior information being acquired continuously during driving, to evaluate crash risk at any time using a measure not dependent on crash occurrence. This method assumes that crash risk is higher in situations that occur more frequently than crashes, and could lead to a rear-end collision (hereinafter referred to as “near-miss situations”) [10], estimating similarity with near-miss situations from the vehicle behavior to deliver a quantitative assessment of crash risk during driving.

First, prior to estimating similarity with near-miss situations, a detector was developed to identify possible near-miss situations from vehicle behavior. The near-miss situation detector was developed in a manner similar to that previously reported by our group [10]. We produced a data set of explanatory variables represented by driver behavior, and the response variable represented as near-miss situations, based on data collected during the participants’ shift. The explanatory variables, vehicle speed and acceleration, were recorded every second by a traffic crash reduction support system (DRIVE CHART, Mobility Technologies, Japan). The response variable was defined as a near-miss situation in which there was a risk of a rear-end collision, which accounts for 53% of reported commercial vehicle crashes [23]. Warnings by the traffic crash reduction support system regarding detected inter-vehicle distance and those by the rear-end collision prevention system (Mobileye570, Mobileye, Israel) were utilized to define near-miss situations that could lead to rear-end collisions. An algorithm to classify the presence or absence of near-miss situations every 20 s from vehicle behavior data was developed by carrying out training and evaluations of response and explanatory variables generated by aggregating the measured speed/acceleration information for every second and the warning alert history in 20 s increments.

The algorithm was developed using a method based on nonparametric analyses [10]. Previous studies have used statistical modeling, that explicitly assumes the mechanism of crash occurrence [17, 20, 21, 33], and analysis based on Bayesian modeling, which is a more flexible method for assuming model structures, such as spatio-temporal structures [18, 34, 35]. However, since the occurrence mechanism of near-miss situations, which are the subject of analysis in the current study, is yet to be adequately elucidated, an exploratory, data-driven method of analyzing the relationship between explanatory and response variables was considered to be effective. Therefore, the analysis was conducted without explicitly assuming the crash occurrence mechanism, by utilizing a gradient boosting decision tree, known to have high classification performance among the nonparametric methods of decision tree methods with high interpretability [10, 36, 37]. The details of the process are given below.

#### Preprocessing

Speed information was used to classify the driving scene for every second. To estimate the appropriate collision risk, road type, one of the road condition factors, is essential [38, 39]. However, due to the limitation of the equipped vehicle sensors in this study, we could not directly record the actual road types, such as expressway driving. Given that the range of driving speed is constrained by the road type [40], we classified the ranges of driving speed (denoted as driving scenes) and training of the detector and classification of crash risk were carried out for data groups classified by scene. Four driving scenes were classified according to the rules shown in Table 1, which were experimentally decided based on the driving speed regulations in Japan [41–43] and the driving speed effects [39, 44]. Each driving scene is as follows: high-speed driving scene such as driving on the expressway, medium-speed driving scene such as driving on ordinary roads, low-speed driving scene such as driving in the premises (e.g., logistic warehouse) of the destination, and extremely low-speed driving scene such as the situation with extremely low or decreased vehicle speed to be able to stop at any time around crossings and intersections.

**Table 1:** Driving scene classification rules.

Driving Scene	Criteria
High-speed	• Cases where speeds of more than 70 km/h occur for more than 30 s, when each time point is aggregated in 1-min units • Even if the above does not apply, cases where the 1-min period prior was determined as “high-speed scene”
Medium-speed	Cases for which none of the above are applicable, traveling at speeds greater than 0 km/hr
Low-speed	Cases where the speed is below 20 km/hr for more than 45 s, continuing for over 4 min, and each time point is aggregated in 1-min units
Extremely low-speed	Cases when at speeds below 3 km/hr, the moving average is below 8 km/hr for approximately 5 s near the time point, and for the 10 s before and after *not applicable to cases with a speed of 0 km/hr

#### Response variable

The response variables were labeled as “1” for situations where the on-board warning of the crash reduction support system or rear-end collision prevention system sounded within 20 s, and 0 for cases where a warning did not sound. To determine whether a situation where the on-board sensor alarm is triggered is truly a high-risk situation, safety transport managers from a logistics company, who were licensed as Operation Manager by the Ministry of Land, Infrastructure, Transport, and Tourism of Japan [45], conducted a visual confirmation of the front-facing video footage from drive records (ND-DVR30, Pioneer, Tokyo, Japan) [46]. Owing to differences in the warning alarm characteristics of the two systems, labeling was conducted with consideration of the differences. The warning alarm system of the inter-vehicle distance alert detected by the on-board sensors of the traffic crash reduction support system was utilized as is, since the Operation Managers confirmed that the system showed high-precision. Meanwhile, due to the low precision and high recall of the rear-end collision alarm sounded by the on-board sensors of the rear-end collision prevention system, the warning alarm used was extracted using a decision tree classifier to adequately extract only the truly high-risk situation alarms from those generated by the same system as previously reported [10].

#### Explanatory variables

Table 2 shows the 15 types of explanatory variables for which 20-s aggregate data were generated from per-second speed and acceleration information.

**Table 2:** Explanatory variables of rear-end collision risk estimation model.

Explanatory Variable Type	Explanatory Variable Name	Description
Maximum speed	max_speed	Maximum speed over 20 s
Minimum speed	min_speed	Minimum speed over 20 s
Average speed	mean_speed	Average speed over 20 s
Maximum directional acceleration	max_acc_x	Maximum directional acceleration over 20 s
Minimum directional acceleration	min_acc_x	Minimum directional acceleration over 20 s
Average directional acceleration	mean_acc_x	Average directional acceleration over 20 s
Maximum lateral acceleration	max_acc_y	Maximum lateral acceleration over 20 s
Minimum lateral acceleration	min_acc_y	Minimum lateral acceleration over 20 s
Average lateral acceleration	mean_acc_y	Average lateral acceleration over 20 s
Speed deviation	std_speed	Speed deviation over 20 s
Directional acceleration deviation	std_acc_x	Directional acceleration deviation over 20 s
Lateral acceleration deviation	std_acc_y	Lateral acceleration deviation over 20 s
Fine speed variation components	std_diff_speed	The 20-s deviation of speed difference with moving average speed (window width 10 s) per second
Average speed difference from the previous 20 s	diff_mspeed_bef	Average speed difference between the average speed of the previous 20 s and the corresponding 20 s
Average speed difference from the following 20 s	diff_mspeed_aft	Average speed difference between the average speed of the following 20 s and the corresponding 20 s

#### Training and evaluation

The model was built using a gradient boosting decision tree utilizing the response and explanatory variables described above [37]. Classification results from the model are produced as a numerical value from 0 to 1. The closer the number is to 1, the higher is the probability of being a near-miss situation. The hyperparameters of the model were determined by a grid search. The classification performance of the near-miss situations was evaluated by the receiver operating characteristic (ROC) curve and area under the ROC curve (AUC) after 5-fold cross validation. In training and evaluation, the low-speed and extremely low-speed scenes were excluded, as they had no near-miss situations relating to rear-end collisions. As a result, the performance in 6649 recordings (approximately 36.9 h) of high-speed scene and that in 9907 recordings (approximately 55.0 h) of medium-speed scene were evaluated (Table 3). To discuss the effects of explanatory variables of the estimated models, we also evaluated the feature importance of the explanatory variables, which represent the contribution of variables [47, 48].

**Table 3:** Training and validation dataset of risk estimation model for each fold.

Driving Scene	Dataset	Risk	Non-Risk	Total
High-speed	Train	997	4655	6649
	Test	172	825	
Medium-speed	Train	200	8221	9907
	Test	36	1450	

#### Risk estimation

Rear-end collision risk, during vehicle operation was estimated over time by using the risk estimation models, with regard to the driving scene, where the classification performance of the near-miss situation was found to be suitable for practical application. The developed models can identify discrete event data indicating the presence or absence of a near-miss situation. However, continuous response variables are preferable when conducting a quantile regression analysis. Since driving behavior is continuous, it can be assumed that collision risk moves continuously pre- and post-near-miss detection according to driving behavior. Based on this assumption, the current research uses the level of near-miss similarity and the detection probability (from 0 to 1) of comprehensible near-miss situations as indicators of collision risk at any given time [49, 50]. Thus, by estimating near-miss situation probability every 20 s using the developed models and the vehicle behavior data obtained continuously while driving, it was possible to quantify the magnitude of collision risk at any time during vehicle operation, without dependence upon crash occurrence.

### Analysis dataset construction

We constructed the analysis dataset by concatenation between the records of the ANF dataset and those of the rear-end collision risk index dataset estimated from their working behaviors dataset. Using the risk estimation model, the rear-end collision risk index was calculated by 20 s based on the explanatory variables obtained from the working behaviors dataset. The estimated risk index was used as an indicator of rear-end collision risk. To match the sampling frequency, the risk index records were resampled from 20-s-resolution to 2-min-resolution by averaging. After resampling, the ANF dataset and the resampled risk index dataset were concatenated. To analyze ANF during driving, we extracted the records when participants drove continuously for 2 min. Additionally, we extracted the records when drivers drove at speeds of 20 km/h or more since the estimated risk index was only validated in the medium-speed and high-speed scenes at speeds of 20 km/h or more. Consequently, we obtained the analysis dataset including 24111 records of approximately 800 working hours of driving situations (Table 4).

**Table 4:** Summary of the amount of analyzed data.

Extraction Condition	All (*n* = 26)	For Analysis (*n* = 20)
Concatenated	279774	219284
Continuous 2 min driving	84116	68006
Over 20km/h term	29327	**24111**

### Analysis of the drivers’ condition indices and crash risk

To analyze whether the drivers’ physiological conditions represented by the ANF indices were associated with the estimated rear-end collision risk index limited from 0 to 100%, we adopted logistic quantile regression analysis [20, 51]. Since high-risk situations during driving rarely occur in occupational drivers, the estimated collision risk distribution was heavily right-tailed, indicating that the conventional ordinary least squares regression and the generalized linear model for the estimation of the average were inadequate. In contrast to the methods to evaluate the mean effects, a quantile regression [52, 53], which does not require any assumptions about the response variable distribution, enables robust and practical estimation of the effects on each quantile even for response variables with skewed distributions. The rear-end collision risk index is a bounded response variable, and, therefore, we employed the logistic quantile regression, which simply uses the logistic transformation of the response variable given the range from *y*_min_ to *y*_max_ [20, 51]. Briefly, in the logistic quantile regression, the conditional *τ*th quantile of the response variable *y*, given the explanatory variables ***X*** as *Q*_*y*_(*τ*), can be described using the following model as

$$Q_{y}(\tau )=\frac{y_{max}exp(X\beta_{\tau })+y_{min}}{1+exp(X\beta_{\tau })}$$

where, the ***β***_*τ*_ represents the regression coefficients of the logistic quantile regression model. Compared to the conditional mean evaluation, the conditional quantile evaluation allows us a wider understanding of the effects of the explanatory variables on the response variable. Methodological details can be referred to in previous studies [20, 51].

Using logistic quantile regression, we analyzed the relationship between ANF and estimated collision risk index after the hierarchical model selection. To evaluate whether the autonomic nervous system (ANS) was associated with the estimated risk, in Step 1, a baseline model only including the control variables (AVGHR, sex, age, and mean speed) that affect ANS [16, 27, 28] was firstly developed. However, the coefficient of sex could not be estimated in this study, as there were no female participants. Next, the models that additionally included the ANF indices were compared. As candidate variables to describe the state of the ANS, in Step 2, we introduced the mainly-SNS-reflecting variable (LF_score_, LF/HF, and SDNN) and the PNS-reflecting variable (HF_score_, NN50, and RMSSD) [32]. To simplify the interpretation of the contributions of SNS and PSN, only the combinations of two variables were compared; interaction terms were not considered in this analysis. For each model, to omit multicollinearity, we checked whether or not the variance inflation factor (VIF) exceeded 10. As the model selection criteria, we calculated Akaike’s information criteria (AIC) [33, 54]. In this analysis, the 25th, 50th, 75th, 90th, and 95th percentiles were evaluated, as the distribution of the estimated collision risk index was heavily right-tailed [20]. In the preliminary analysis, the minimum-AIC models were different depending on the quantiles. To discuss the relationship by the same structure model, we selected the model based on the average rank of AIC in each quantile and the significance of the estimated coefficients introduced in Step 2. The standard errors of the estimated coefficients were obtained based on bootstrapping with 2000 times resampling. After model selection, we evaluated the quantile and the mean effects of the explanatory variables on the estimated collision risk based on the obtained model using logistic quantile regression and logistic regression, respectively.

### Evaluation of the drivers’ state-dependent ANF difference

To clarify the effects of the truck drivers’ work on their physiological conditions, we analyzed the difference between the mid-shift ANF and the pre- and post-shift ANFs. To evaluate the effect of starting their work shifts, in the mid-shift, the ANF indices averaged by 30 min immediately after the initiation of driving work were used as the representative values during driving work. As the pre- and post-shift indices, we used the ANF indices obtained in the drivers’ resting eye-closing state before starting and after finishing their work shifts, respectively. We compared the ANF indices for each timing except for time-domain features (SDNN, NN50, and RMSSD) due to the system limitation in pre- and post-shift. In this analysis, the ANF differences were compared using Tukey-Kramer’s test if the dataset were found to be normally distributed based on the Shapiro-Wilk test; otherwise, we used the Steel–Dwass test.

### Statistical analysis

All data processing and analyses were performed using Python 3.6 including SciPy 1.0 and scikit-learn 0.18. The logistic quantile regression analysis and the multiple comparison tests were performed using R 3.6 including quantreg 5.54, multcomp 1.4, and NSM3 1.15. *p*<0.05 was considered to be statistically significant. Statistical significance is denoted as *p<0.05, **p<0.01, and ***p<0.001.

## Results

### Evaluation of estimated collision risk during driving

**Fig 2:**
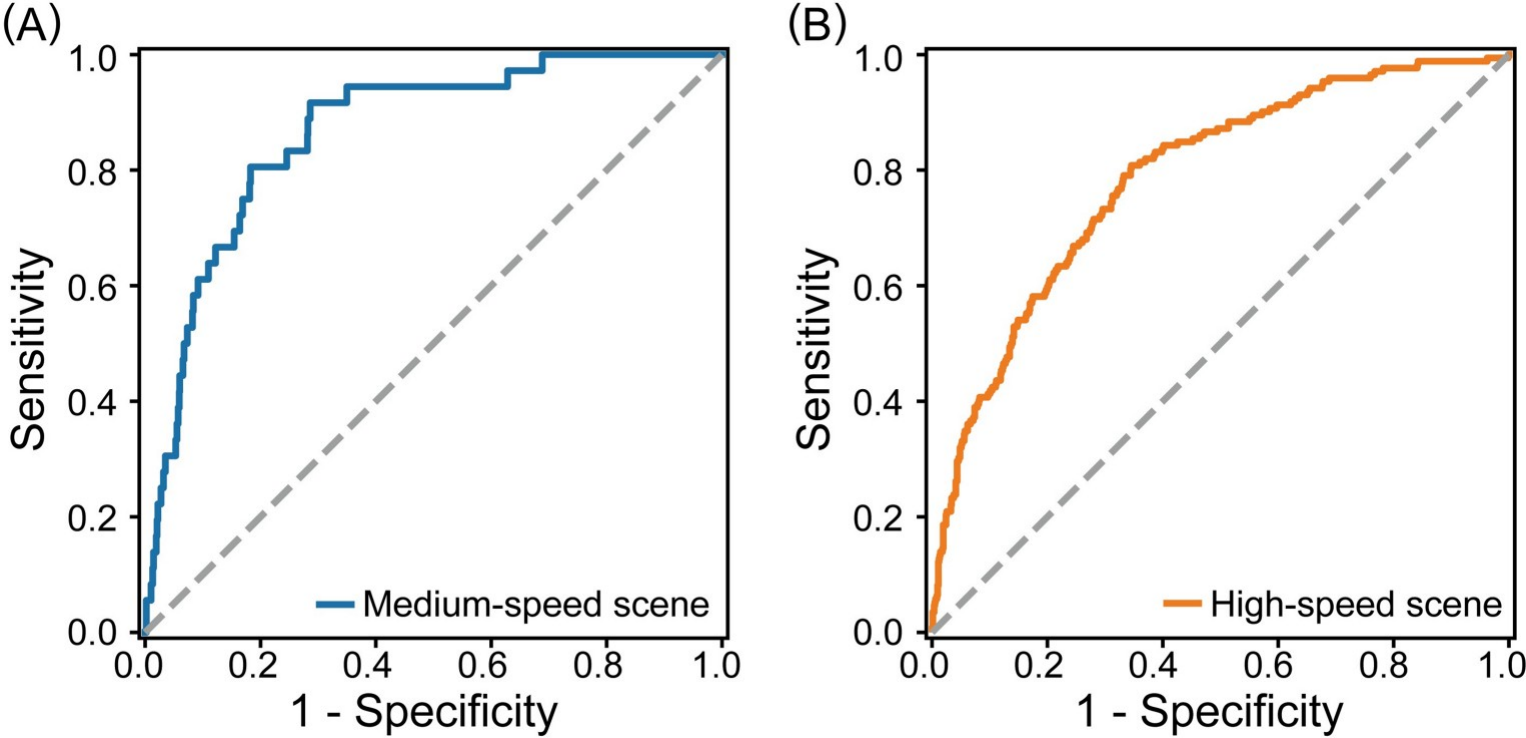
ROC curve analysis for discriminating near-miss situations. (A) ROC curve of the risk estimation model for medium-speed scenes (AUC = 0.867). The dotted gray line depicts the ROC curve whose AUC = 0.500 for reference. (B) ROC curve of the risk estimation model for high-speed scenes (AUC = 0.787).

The classification performance of the detector developed to assess collision risk over time in near-miss situations that could potentially lead to a crash was evaluated. Of the four driving scenes, we only evaluated the performance for high-speed and medium-speed scenes, excluding low-speed and extremely low-speed scenes due to lack of near-miss situations related to rear-end collision. The performances were evaluated in terms of AUC by ROC curve analysis (Fig 2). Results of the AUC evaluation of the model trained on each scene using this data set were: AUC = 0.787 for high-speed scenes and AUC = 0.867 for medium-speed scenes. These results confirm that the classification performance is sufficient for the evaluation of collision risk over time, as the high-speed scene had acceptable discrimination and the medium-speed scene had excellent discrimination [55].

Next, we showed the contribution of the resulting model’s explanatory variables (see S1 Table for detailed results). In the high-speed model, the contribution of the explanatory variables was at most approximately 9% and at least approximately 5%. There was no large bias observed in contribution among the explanatory variables. Five explanatory variables with contributions above 7% were maximum speed (approximately 9%), average speed (approximately 9%), average speed difference from the following 20 s (approximately 8%), average speed difference from the previous 20 s (approximately 7%), and maximum directional acceleration (approximately 7%). Moreover, in the medium-speed model, the largest contribution of the explanatory variable when driving on local roads was approximately 14%, and the lowest contribution was approximately 5%, and no large bias found. Explanatory variables greater than 7% were maximum speed (approximately 8%), average speed (approximately 10%), and minimum speed (approximately 14%).

### Relationship between drivers’ condition indices and crash risk

**Fig 3:**
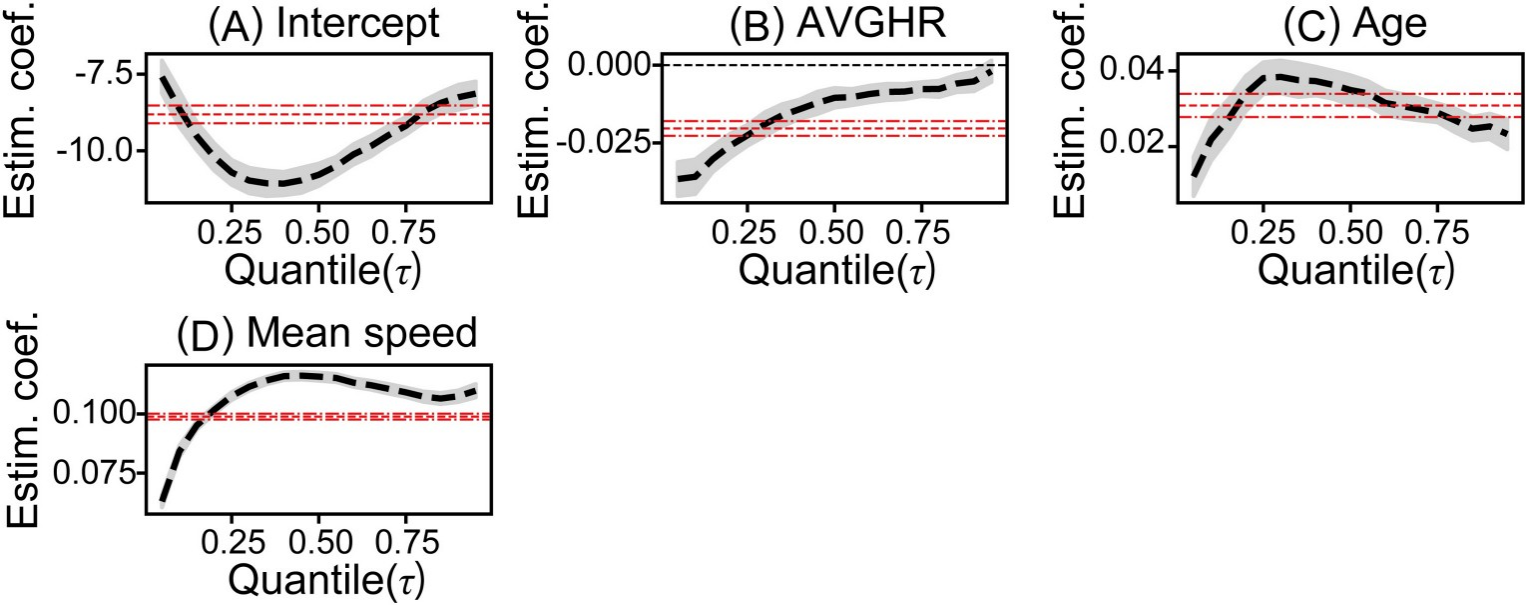
Estimated coefficients of the baseline model. Coefficients over each quantile of (A) Intercept, (B) average heart rate, AVGHR, (C) Age, (D) Mean speed. Black dashed line shows estimated coefficients, and gray shaded area depicts bootstrapping 95% confidence interval. Red dashed lines show the coefficient of the logistic regression model and its 95% confidence interval.

**Fig 4:**
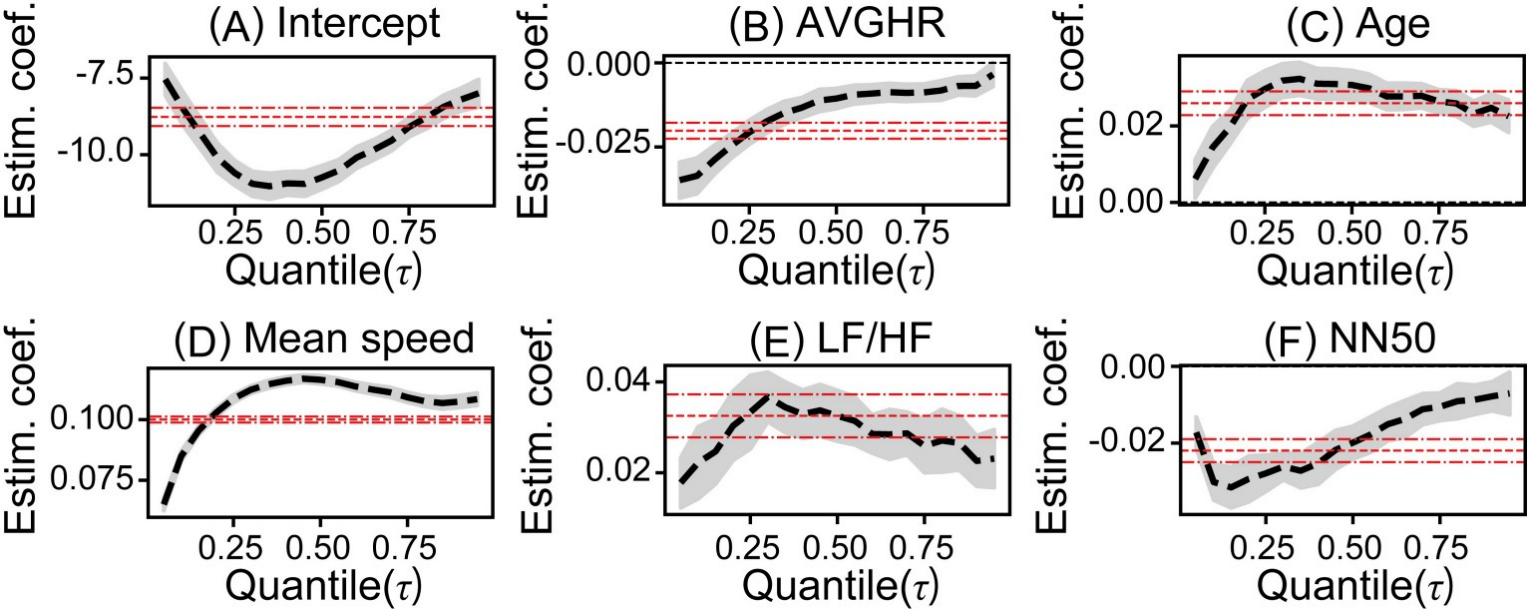
Estimated coefficients of the selected model 5. Coefficients over each quantile of (A) Intercept, (B) average heart rate, AVGHR, (C) Age, (D) Mean speed, (E) LF/HF, (F) NN50. Black dashed line shows estimated coefficients, and gray shaded area depicts bootstrapping 95% confidence interval. Red dashed lines show coefficient of logistic regression model and its 95% confidence interval.

To analyze the relationship between estimated collision risk and ANF when driving, we first conducted a hierarchical model selection based on AIC for each model of the ANF-based crash risk analysis model using logistic quantile regression. Compared to the baseline model built in Step 1, the estimation results for control variables in the Step 2 models 1–9 were generally consistent (e.g., Fig 3 vs. Fig 4), and improvements in AIC were observed in all models (Table 5). These results suggest that the ANF index is an effective variable for analysis of estimated collision risk during driving. Next, in a comparison of the Step 2 models, the estimation results of the coefficients estimated for each quantile corresponding to each SNS and PNS variable were roughly equivalent between the models (e.g., Fig 3 vs. S1 Fig). Meanwhile, the model with the smallest AIC differed depending on the quantile (Table 5). To analyze the relationships in a single model, results of an average rank calculation based on AIC, indicated that model 5 had the lowest average rank. In addition, results from an examination of the significance of the variable pairs entered in Step 2 indicated that the coefficients of the entered variables were all significant for models 1–3 and model 5. Based on the above results, model 5 was selected and evaluated as an ANF-based crash risk analysis model.

**Table 5:** AIC-based hierarchical selection of estimated rear-end collision risk analysis model.

	#	Model	*τ* = 0.25	*τ* = 0.50	*τ* = 0.75	*τ* = 0.90	*τ* = 0.95	Avg. rank
Step 1	*	AVGHR + Age + Mean speed	106072.8	99351.4	98438.8	102445.5	106337.1	–
Step 2	1	**LF**_**score**_ **+ HF**_**score**_ **+ AVGHR + Age + Mean speed**	105668.9	98994.5	**98198.6**	102260.5	106152.6	3.6
	2	LF_score_ + NN50 + AVGHR + Age + Mean speed	**105364.8**	**98967.2**	98262.8	102359.8	106272.5	4.6
	3	LF_score_ + RMSSD + AVGHR + Age + Mean speed	105523.0	99012.2	98272.7	102353.7	106280.4	5.6
	4	LF/HF + HF_score_ + AVGHR + Age + Mean speed	105797.1	99066.4	98213.2	102243.3	**106130.8**	4.8
	5	**LF/HF + NN50 + AVGHR + Age + Mean speed**	105487.9	**98916.8**	**98158.3**	**102221.4**	**106122.4**	1.4
	6	LF/HF + RMSSD + AVGHR + Age + Mean speed	105704.2	99022.8	**98201.6**	**102242.5**	106138.7	4.0
	7	SDNN + HF_score_ + AVGHR + Age + Mean speed	105938.2	99234.3	98363.4	102354.1	106242.4	7.4
	8	SDNN + NN50 + AVGHR + Age + Mean speed	**105483.4**	99065.8	98337.6	102369.6	106256.9	5.8
	9	SDNN + RMSSD + AVGHR + Age + Mean speed	105687.9	99150.3	98370.1	102381.0	106269.7	7.8

Underlined bold and bold text represent smallest and second smallest-AIC model in each quantile, respectively.

Tables 6 and 7 depict estimate results of the baseline model and the selected crash risk analysis model (see S2 Table for further results). First, when comparing the baseline model with the selected crash risk analysis model 5, the results of estimation for each quantile of the control variables were found to be roughly consistent (Fig 3 vs. Fig 4). Moreover, for the control variable of AVGHR, heart rate increases tended to reduce the estimated risk of crashes, particularly at lower quantiles (Fig 4B). Age was shown to increase estimated collision risk (Fig 4C). Similarly, for mean vehicle speed, estimated collision risk increased as mean vehicle speed increased, and the effect of all the quantiles shown in Table 6 was greater than the mean effect (Fig 4D).

**Table 6:** Estimated coefficients of the baseline model by logistic quantile regression and logistic regression.

Variable		*τ* = 0.25	*τ* = 0.50	*τ* = 0.75	*τ* = 0.90	*τ* = 0.95	Mean
Intercept	Estim.	-10.707*	-10.783*	-9.185*	-8.255*	-8.135*	-8.811*
	SE	0.206	0.185	0.148	0.207	0.201	0.147
AVGHR	Estim.	-0.023*	-0.010*	-0.008*	-0.005*	-0.002	-0.020*
	SE	0.002	0.002	0.001	0.002	0.002	0.001
Age	Estim.	0.038*	0.035*	0.029*	0.025*	0.023*	0.031*
	SE	0.002	0.002	0.001	0.002	0.002	0.002
Mean speed	Estim.	0.108*	0.116*	0.109*	0.107*	0.110*	0.099*
	SE	0.001	0.001	0.001	0.001	0.001	0.001

Mean (SE with 2000 samples bootstrapping)

*p<0.05

**Table 7:** Estimated coefficients of the selected model 5.

Variable		*τ* = 0.25	*τ* = 0.50	*τ* = 0.75	*τ* = 0.90	*τ* = 0.95	Mean
Intercept	Estim.	-10.598*	-10.739*	-9.097*	-8.198*	-7.993*	-8.765*
	SE	0.207	0.194	0.161	0.189	0.228	0.153
LF/HF	Estim.	0.033*	0.033*	0.026*	0.023*	0.023*	0.033*
	SE	0.004	0.003	0.003	0.003	0.003	0.002
NN50	Estim.	-0.028*	-0.02*	-0.011*	-0.008*	-0.007*	-0.022*
	SE	0.002	0.002	0.002	0.002	0.003	0.002
AVGHR	Estim.	-0.021*	-0.011*	-0.009*	-0.007*	-0.003	-0.02*
	SE	0.002	0.002	0.001	0.001	0.002	0.001
Age	Estim.	0.030*	0.031*	0.026*	0.025*	0.023*	0.026*
	SE	0.002	0.002	0.002	0.002	0.002	0.002
Mean speed	Estim.	0.109*	0.116*	0.109*	0.107*	0.108*	0.100*
	SE	0.001	0.001	0.001	0.001	0.001	0.001

Mean (SE with 2000 samples bootstrapping)

*p<0.05

Lastly, the effect of the ANF index was examined using the selected crash risk analysis model (Table 7). First, it was confirmed that estimated collision risk increased along with increases in LF/HF (Fig 4E). Since LF/HF is an indicator of sympatho-vagal balance [56], this suggests an increased estimated collision risk with activation of sympathetic nerve activity. This effect was smaller than the effect on mean values in both tails of the distribution. In addition, the estimated collision risk was observed to be reduced as NN50 increased (Fig 4F). Given that NN50 is an indicator of PNS activity [32], this suggests a tendency for decreased estimated collision risk as parasympathetic nerve activity activates. This effect was found to be larger than the effect on the mean values at the lower quantiles below the median. Even in other candidate models of the model selection Step 2 (Table 5), a similar relationship between the ANF indicators and collision risks was also observed (e.g. the model 1 using not LF/HF and NN50 but LF_score_ and HF_score_, S1 Fig, S2 and S3 Tables)

### Effects of driving work shift on drivers’ condition

**Fig 5:**
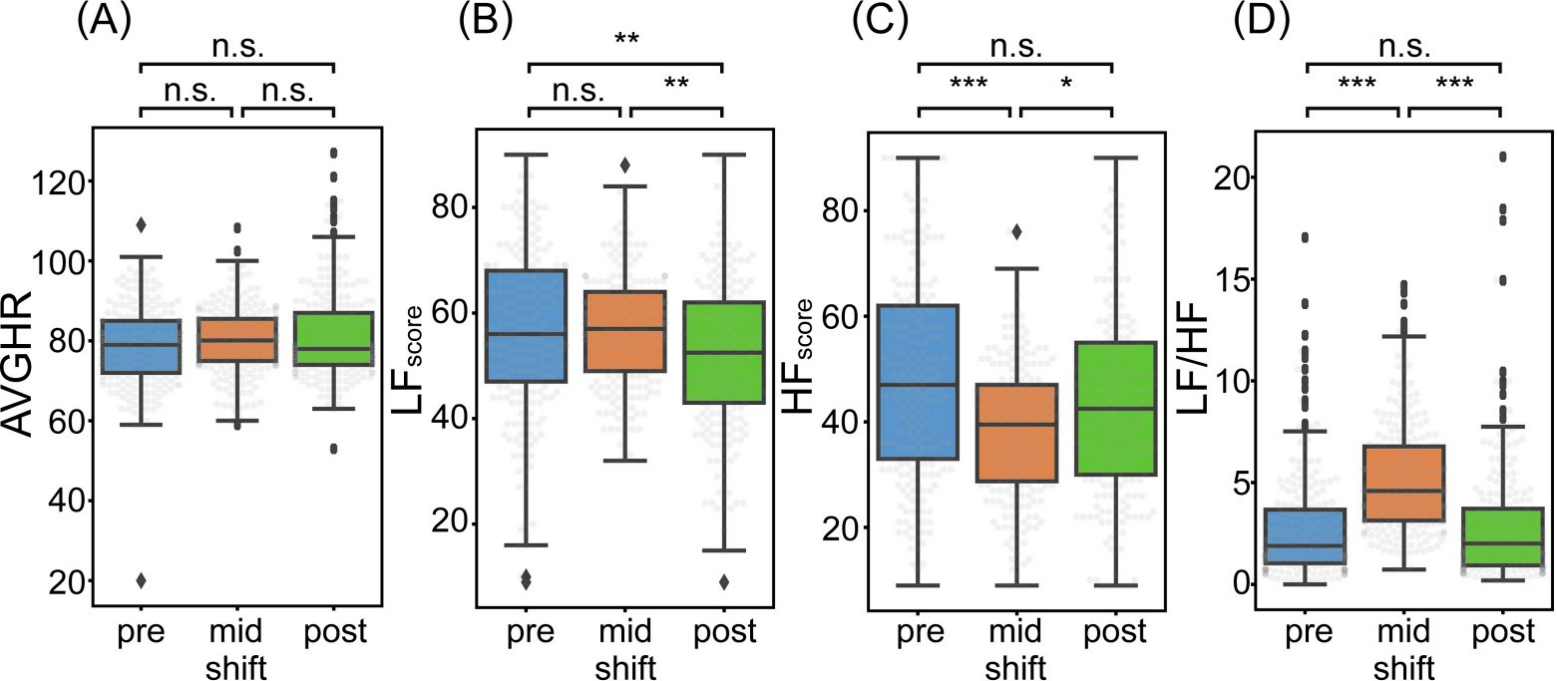
Comparison between ANF of resting eye-closing state in pre-shift (left), driving state immediately after starting driving in mid-shift (middle), and resting eye-closing state in post-shift (right). (A) average heart rate, AVGHR; (B) devised score of LF, LF_score_; (C) devised score of HF, HF_score_; (D) LF/HF. *p<0.05, **p<0.01, and ***p<0.001 by Tukey–Kramer’s test or Steel–Dwass’s test.

Differences in physiological condition while driving were assessed, specifically immediately after commencing driving, and in a resting state with drivers’ eyes closed pre- and post-shift, to assess the impact of truck driving on physiological condition (Fig 5, Table 8). Firstly, there were no significant differences in AVGHR for any pair (Fig 5A). Moreover, a comparison between pre-shift and immediately post commencement showed no significant physiological differences in LF_scores_ (Fig 5B). In contrast, HF_score_ decreased significantly after driving commencement (Fig 5C). In addition, LF/HF increased significantly after driving commencement (Fig 5D). Since HF and LF/HF are indicators of PNS activity and sympatho-vagal balance, respectively [31, 56], and HF_score_ is a devised score of HF, reduction in PNS activity when driving compared to the resting eyes-closed condition before work, indicates that SNS activity was relatively dominant.

**Table 8:** Difference of ANF between resting eye-closing state in pre-shift, driving state immediately after starting driving in mid-shift, and resting eye-closing state in post-shift.

ANF	Method	*p*-value		
		ANF_mid_-ANF_pre_	ANF_post_-ANF_pre_	ANF_post_-ANF_mid_
AVGHR	Steel–Dwass	0.160	0.126	0.999
LF_score_	Tukey–Kramer	0.958	0.009**	0.004**
HF_score_	Steel–Dwass	<0.001***	0.051	0.012*
LF/HF	Steel–Dwass	<0.001***	0.928	<0.001***

*p<0.05

**p<0.01, and

***p<0.001 by Tukey–Kramer’s test or Steel–Dwass’s test.

Lastly, we compared the physiological conditions before, during, and after work. HF_score_ and LF/HF were confirmed to be significantly higher and lower, respectively, before driving than when driving and recovered to pre-shift levels after work (Fig 5C and 5D). In contrast, differences were found between pre- and mid-shift LF_scores_, indicating a significant decrease when compared with either situation (Fig 5B). These results indicate that the relatively dominant state of the SNS observed while driving dissipates when driving ceases, and SNS activity decreases compared to other time points.

## Discussion

In this study, we developed a collision risk index that estimated the magnitude of the risk of a rear-end collision from vehicle behavior alone, to quantify collision risk while driving, even in the absence of a traffic crash. An analysis of the relationship between the estimated collision risk indicator and ANF during actual operation of a vehicle revealed that an increase in sympathetic nerve activity leads to an increase in collision risk indicators, whereas an increase in parasympathetic nerve activity leads to a decrease in collision risk indicators. The above results suggest that ANF assessment during vehicle operation is useful in reducing collision risk while driving.

We developed a model for estimating rear-end collision risk by estimating the risk indicators of rear-end collision based on vehicle behavior. The contribution of the explanatory variables adopted in the resulting model was analyzed and speed-related exploratory variables were incorporated throughout. Additionally, the explanatory variables of acceleration were also included in the higher rank for high-speed scene. Since rapid acceleration and deceleration are directly related to high-risk in high-speed scene, low acceleration values are considered likely when driving with low-risk. In contrast, in near-miss situations, acceleration may increase in accordance with temporary acceleration and deceleration [57]. It is considered that the explanatory variable of acceleration included in high-speed scene reflects the previously mentioned characteristics of high-speed scene. Consequently, it is thought that with the addition of acceleration, risky-situations can be better captured compared to previous studies that only used speed and alarm sounding duration [10]. However, our risk estimation has several limitations. First, due to the equipped sensors’ limitation, we indirectly considered the effects of road types on collision risks from vehicle speed range in the driving scene classification [38, 39]. In future studies, the utilization of continuous video monitoring and light detection and ranging (LiDAR) sensors will provide richer surrounding information, including road types [58, 59]. Second, since driving environment effects, such as time of day, weather, and road conditions, have also been reported as crash-related factors [34, 35, 38], further expansion of explanatory variables in the future will improve the existing knowledge on the topic. Third, since the inter-vehicle gap and rear-end collision warnings used as rear-end collision risks in this study are reported in the speed ranges of typical driving conditions on public roads, they have difficulty sounding before and after the vehicle stops and at speeds below 20 km/hr. Therefore, the model developed in this study for estimating rear-end collision risk was unable to estimate crash risk in cases of low-speed driving, and extremely low-speed driving scenes at low speeds. Future research should define and analyze crash risk at low speeds based on a different warning system and method.

The crash risk analysis model in this study was built through hierarchical model selection. When compared with the coefficients of the control variables in the baseline model created in Step 1, the estimation result of the control variables in model 5, selected in Step 2, is similar, and the estimation of the selected model is considered likely to be successful. Furthermore, several findings were obtained from the coefficients of the control variables. First, age effects indicated an increased estimated collision risk with increasing age. The results are thought to support these findings given that physical, sensory, and cognitive impairments that occur with age, such as decline in vision and attention, are known to affect driving ability [60]. Moreover, in our crash risk analysis model, average vehicle speed increases contributed to the increase in the estimated collision risk. These results are consistent with the previous finding that driving speed effects impact crash rate by an exponential function and a power function [39]. Since average vehicle speed exhibits a large effect compared to other variables, and the risk associated with the increase in the average vehicle speed is high, the estimated rear-end collision risk index developed in this study mainly captures risks occurring at high speeds.

The relationship between driver’s physiological condition and rear-end collision risk was revealed by the addition of such elements to the crash risk analysis model that indicate the ANF of the driver. The selected crash risk analysis model 5 suggested a tendency of increased estimated risk of collision with the activation of the drivers’ sympathetic nerve activity indicated by increased LF/HF and inhibition of parasympathetic nerve activity as indicated by decreased NN50. Increased LF/HF has generally been associated with acute stress and fatigue [56, 61]. Moreover, it is also known that heart rate variability (HRV) decreases during stress loads such as occupational stress, and indicators of PNS activity, NN50, pNN50, RMSSD, and HF, decrease [56, 62]. Based on the above, ANF indicators in the crash risk analysis model may reflect the physiological state in the over-arousal state [13], suggesting that acute stress-induced fatigue increases the risk of rear-end collision.

Based on the obtained results, we can consider the possible mechanisms of stress-induced fatigue on crash risk and crash prevention measures. A previous questionnaire-based study reported that stressful working conditions predict high-risk behavior in bus rapid transport drivers mediated by fatigue [7], which supports our result, although the time scales on which both phenomena focus are different. Other studies also clarify that the fatigued situation degrades cognitive and motor performance, such as inattention and decreased reaction to dangerous situations [5, 6]. Even in ANF indicators, increased SNS activity and decreased PNS activity are associated with the worse cognitive performance [63]. Considering this knowledge, the potential mechanism by which stress-induced fatigue associates with collision risk can be interpreted as follows: fatigue induced by stressful situations causes low mental and physical performance. The poor performance subsequently makes it difficult to maintain appropriate inter-vehicle distance, which results in an increased collision risk, and can finally cause subsequent crashes. Assuming this mechanism, stress and fatigue management strategies based on ANF during driving could help reduce traffic crashes. If a mid-driving ANF monitoring system will be developed, allowing for the detection of signs of stress or fatigue, then methods available while driving (e.g., listening to healing music [64]) and those possible to practice at other times (e.g., performing yoga [65], Zen meditation [66]) can be expected to contribute to the reduction of traffic crashes caused by poor physiological conditions, via ANF improvement.

While ANF indicators show the physiological response seen in over-arousal state, the AVGHR response also requires consideration. These results showed that the risk of crashes was reduced with an increase in AVGHR, which is the opposite of the physiological response reported in the over-arousal state [13]. A decrease in AVGHR has been reported as a strong indicator of drowsiness [67]. In addition, under-arousal states such as increased drowsiness, disengagement, and decreased vigilance [68] are reported to be accompanied by decreased heart rates [13]. Considering these results, the estimated rear-end collision risk analysis model implies that the ANF indices reflect the risk related to the over-arousal state and AVGHR reflects the risk related to the under-arousal state. In the future, comparative analysis of crash risk indicators that reflect under-arousal, such as drowsiness, as the response variable, will provide an even broader understanding of the relationship between crash risk and physiological condition.

Finally, based on a comparative evaluation of ANF while driving, and before and after driving, the necessity of physiological measurement while driving was examined. Commencement of driving was found to be accompanied by inhibition in PNS activity, leading to a relative SNS dominance, and this state was resolved upon cessation of driving. This result is consistent with previous research on SNS dominance while driving [13]. Even when comparing temporally close conditions, pre-shift and immediately after commencement of work, significant differences in the state of the ANS are indicated. In addition, when pre-and post-shift are compared under the same measurement conditions, changes in physiological condition due to driving, such as a significant decrease in LF_score_, were observed. Furthermore, the state of the ANS changes over time due to factors such as the circadian rhythm [69]. Considering the dynamic changes outlined above, when carrying out traffic crash countermeasures based on physiological condition, it may be possible to expand the scope of measures by combining the evaluation of the physiological condition before and after work with the evaluation of the physiological condition while driving.

Although our results showed that, monitoring the drivers’ ANS while driving is important for the evaluation of collision risk, this study has some limitations. First, as the participants were mainly mature males, gender and age differences have not been fully evaluated. In the future, there is a need to evaluate a more heterogenous sample. Second, compared with previous studies, the effect of the variables and interaction terms has not been considered. The relationship between the two could be further evaluated by analysis with an extended mixed model, such as logistic transformation of linear quantile mixed model, which incorporates random effects [18, 22, 33]. Finally, the causal relationship between collision risk and physiological condition and predictability could not be evaluated in this study. By analyzing causal relationships and predictability, including the expansion of the analysis model to a time-series model and considering temporal correlation, it may be possible to detect crash risk and implement effective measures to avoid it [70, 71].

## Conclusion

This study aimed to clarify the relationship between drivers’ physiological condition in terms of ANF and an indicator of rear-end collision risk in on-road driving situations. Our results demonstrated that activation of sympathetic nerve activity and inhibition of parasympathetic nerve activity increased each quantile of the rear-end collision risk index. This suggests that during driving in actual on-road situations, acute stress-induced drivers’ fatigue increases rear-end collision risk. Our findings emphasize the importance of truck drivers’ physiological condition monitoring even in mid-shift to prevent rear-end collisions. Therefore, the development of the ANF-based stress warning and relief system using drivers’ continuous monitoring could contribute to the prevention of a broader range of road crashes including rear-end collisions. Further studies on predicting the increasing risk caused by drivers’ condition changes will help to promote safe driving.

## Supporting information

S1 Fig Estimated coefficients of model 1. Coefficients over each quantile of (A) Intercept, (B) average heart rate, AVGHR, (C) Age, (D) Mean speed, (E) LF_score_, (F) HF_score_. Black dashed line shows estimated coefficients and gray shaded area depicts bootstrapping 95% confidence interval. Red dashed lines show the coefficient of logistic regression model and its 95% confidence interval.
(TIF)

S1 Table Contribution of each explanatory variable in risk estimation model. (DOCX)

S2 Table Detailed parameters of baseline model, model 1, and model 5 by logistic quantile regression and logistic regression. (DOCX)

S3 Table Estimated coefficients of model 1. (DOCX)
