# Supplementary figures and images for: Increase in rear-end collision risk by acute stress-induced fatigue in on-road truck driving
Source: PLoS One. 2021 Oct 21;16(10):e0258892. doi: 10.1371/journal.pone.0258892 (PMC8530353; doi:10.1371/journal.pone.0258892)

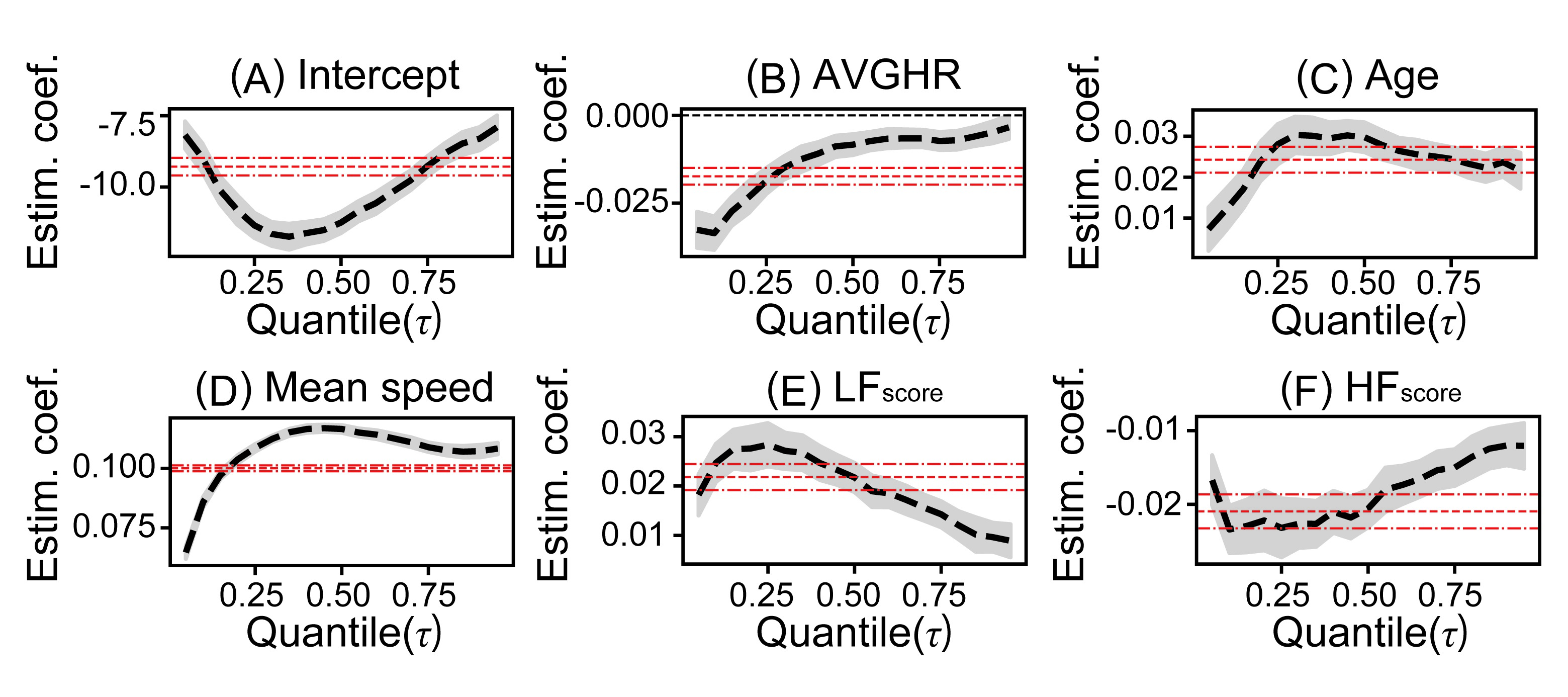

Supplement: S1 Fig — Coefficients over each quantile of (A) Intercept, (B) average heart rate, AVGHR, (C) Age, (D) Mean speed, (E) LFscore, (F) HFscore. Black dashed line shows estimated coefficients and gray shaded area depicts bootstrapping 95% confidence interval. Red dashed lines show the coefficient of logistic regression model and its 95% confidence interval. (TIF) [file pone.0258892.s001.tif]
