# Supplementary material for: Increase in rear-end collision risk by acute stress-induced fatigue in on-road truck driving
Source: PLoS One. 2021 Oct 21;16(10):e0258892. doi: 10.1371/journal.pone.0258892 (PMC8530353; doi:10.1371/journal.pone.0258892)
Supplement: S1 Table — (DOCX) [file pone.0258892.s002.docx]

**S1 Table. Contribution of Each Explanatory Variable in Risk Estimation Model.**

| **Explanatory Variable Name** | **Feature Importance in High-speed model [%]** | **Feature Importance in Medium-speed model [%]** |
| --- | --- | --- |
| max_speed | 9.34 | 8.02 |
| min_speed | 6.27 | 13.96 |
| mean_speed | 8.58 | 9.79 |
| max_acc_x | 7.13 | 5.59 |
| min_acc_x | 6.77 | 5.74 |
| mean_acc_x | 5.43 | 5.52 |
| max_acc_y | 5.45 | 4.59 |
| min_acc_y | 5.38 | 5.59 |
| mean_acc_y | 6.42 | 5.27 |
| std_speed | 6.47 | 5.73 |
| std_acc_x | 6.67 | 6.26 |
| std_acc_y | 5.65 | 5.92 |
| std_diff_speed | 5.68 | 6.40 |
| diff_mspeed_bef | 7.15 | 5.52 |
| diff_mspeed_aft | 7.61 | 6.09 |
