# Supplementary material for: Increase in rear-end collision risk by acute stress-induced fatigue in on-road truck driving
Source: PLoS One. 2021 Oct 21;16(10):e0258892. doi: 10.1371/journal.pone.0258892 (PMC8530353; doi:10.1371/journal.pone.0258892)
Supplement: S2 Table — (DOCX) [file pone.0258892.s003.docx]

**S2 Table. Detailed Parameters of Baseline Model, Model 1, and Model 5
by Logistic Quantile Regression and Logistic Regression.**

| **Model #** | **Variable** | | ***τ* = 0.1** | ***τ* = 0.25** | ***τ* = 0.5** | ***τ* = 0.75** | ***τ* = 0.9** | ***τ* = 0.95** | **Mean** |
| --- | --- | --- | --- | --- | --- | --- | --- | --- | --- |
| * | log pseudo-likelihood | | -57334.3 | -53032.4 | -49671.7 | -49215.4 | -51218.7 | -53164.5 | -49145.7 |
|  | *ρ*^2^ | | 0.352 | 0.376 | 0.331 | 0.316 | 0.261 | 0.233 | 0.506 |
|  | Intercept | *t* | -29.123 | -52.038 | -58.396 | -62.259 | -39.955 | -40.489 | -59.753 |
|  |  | *p* | <0.001 | <0.001 | <0.001 | <0.001 | <0.001 | <0.001 | <0.001 |
|  | AVGHR | *t* | -12.670 | -13.205 | -6.543 | -6.805 | -3.240 | -1.111 | -16.814 |
|  |  | *p* | <0.001 | <0.001 | <0.001 | <0.001 | 0.001 | 0.267 | <0.001 |
|  | Age | *t* | 7.644 | 17.862 | 17.575 | 22.788 | 14.784 | 11.426 | 19.733 |
|  |  | *p* | <0.001 | <0.001 | <0.001 | <0.001 | <0.001 | <0.001 | <0.001 |
|  | Mean speed | *t* | 68.357 | 127.688 | 134.473 | 108.029 | 87.033 | 77.044 | 155.947 |
|  |  | *p* | <0.001 | <0.001 | <0.001 | <0.001 | <0.001 | <0.001 | <0.001 |
| **Model #** | **Variable** | | ***τ* = 0.1** | ***τ* = 0.25** | ***τ* = 0.5** | ***τ* = 0.75** | ***τ* = 0.9** | ***τ* = 0.95** | **Mean** |
| 1 | log pseudo-likelihood | | -57137.3 | -52828.5 | -49491.3 | -49093.3 | -51124.2 | -53070.3 | -48970.8 |
|  | *ρ*^2^ | | 0.358 | 0.380 | 0.335 | 0.321 | 0.264 | 0.236 | 0.513 |
|  | Intercept | *t* | -34.641 | -50.505 | -59.582 | -52.710 | -40.181 | -37.515 | -58.991 |
|  |  | *p* | <0.001 | <0.001 | <0.001 | <0.001 | <0.001 | <0.001 | <0.001 |
|  | LF_score_ | *t* | 12.582 | 12.653 | 14.399 | 10.588 | 6.447 | 5.225 | 16.161 |
|  |  | *p* | <0.001 | <0.001 | <0.001 | <0.001 | <0.001 | <0.001 | <0.001 |
|  | HF_score_ | *t* | -14.280 | -11.384 | -15.847 | -11.849 | -8.771 | -7.656 | -17.824 |
|  |  | *p* | <0.001 | <0.001 | <0.001 | <0.001 | <0.001 | <0.001 | <0.001 |
|  | AVGHR | *t* | -13.856 | -9.942 | -5.519 | -5.616 | -3.106 | -2.012 | -14.212 |
|  |  | *p* | <0.001 | <0.001 | <0.001 | <0.001 | 0.002 | 0.044 | <0.001 |
|  | Age | *t* | 4.630 | 11.182 | 16.035 | 16.650 | 12.964 | 9.636 | 15.046 |
|  |  | *p* | <0.001 | <0.001 | <0.001 | <0.001 | <0.001 | <0.001 | <0.001 |
|  | Mean speed | *t* | 75.340 | 133.787 | 142.589 | 107.796 | 80.895 | 88.861 | 157.841 |
|  |  | *p* | <0.001 | <0.001 | <0.001 | <0.001 | <0.001 | <0.001 | <0.001 |

Baseline model *, AVGHR + Age + Mean speed; Model 1, LF_score_ + HF_score_ + AVGHR + Age + Mean speed; ^*^log pseudo-likelihood and *ρ*^2^ in Logistic regression model in Column Mean represent log likelihood and adjusted-*R*^2^, respectively; *t* and *p* represent *t*-value and *p*-value with 2000 samples bootstrapping, respectively.

**S2 Table. Continued.**

| **Model #** | **Variable** | | ***τ* = 0.1** | ***τ* = 0.25** | ***τ* = 0.5** | ***τ* = 0.75** | ***τ* = 0.9** | ***τ* = 0.95** | **Mean** |
| --- | --- | --- | --- | --- | --- | --- | --- | --- | --- |
| 5 | log pseudo-likelihood | | -57005.0 | -52738.0 | -49452.4 | -49073.2 | -51104.7 | -53055.2 | -48902.3 |
|  | *ρ*^2^ | | 0.362 | 0.383 | 0.337 | 0.321 | 0.265 | 0.236 | 0.516 |
|  | Intercept | *t* | -30.573 | -51.304 | -55.386 | -56.441 | -43.263 | -35.061 | -57.292 |
|  |  | *p* | <0.001 | <0.001 | <0.001 | <0.001 | <0.001 | <0.001 | <0.001 |
|  | LF/HF | *t* | 5.507 | 8.086 | 11.435 | 8.657 | 7.964 | 7.133 | 13.471 |
|  |  | *p* | <0.001 | <0.001 | <0.001 | <0.001 | <0.001 | <0.001 | <0.001 |
|  | NN50 | *t* | -12.704 | -11.616 | -9.144 | -5.813 | -4.069 | -2.511 | -14.383 |
|  |  | *p* | <0.001 | <0.001 | <0.001 | <0.001 | <0.001 | 0.012 | <0.001 |
|  | AVGHR | *t* | -12.500 | -11.398 | -7.050 | -7.000 | -4.606 | -1.905 | -16.666 |
|  |  | *p* | <0.001 | <0.001 | <0.001 | <0.001 | <0.001 | 0.057 | <0.001 |
|  | Age | *t* | 5.163 | 12.219 | 16.021 | 17.060 | 13.095 | 10.497 | 16.369 |
|  |  | *p* | <0.001 | <0.001 | <0.001 | <0.001 | <0.001 | <0.001 | <0.001 |
|  | Mean speed | *t* | 84.422 | 135.121 | 136.919 | 103.264 | 87.240 | 83.497 | 158.678 |
|  |  | *p* | <0.001 | <0.001 | <0.001 | <0.001 | <0.001 | <0.001 | <0.001 |

Model 5, LF/HF + NN50 + AVGHR + Age + Mean speed.
