# Supplementary material for: Increase in rear-end collision risk by acute stress-induced fatigue in on-road truck driving
Source: PLoS One. 2021 Oct 21;16(10):e0258892. doi: 10.1371/journal.pone.0258892 (PMC8530353; doi:10.1371/journal.pone.0258892)
Supplement: S3 Table — (DOCX) [file pone.0258892.s004.docx]

**S3 Table.** **Estimated Coefficients of Model 1.**

| **Variable** | | ***τ* = 0.25** | ***τ* = 0.50** | ***τ* = 0.75** | ***τ* = 0.90** | ***τ* = 0.95** | **Mean** |
| --- | --- | --- | --- | --- | --- | --- | --- |
| Intercept | Estim. | -11.347^*^ | -11.247^*^ | -9.258^*^ | -8.303^*^ | -7.903^*^ | -9.284^*^ |
|  | SE | 0.225 | 0.189 | 0.176 | 0.207 | 0.211 | 0.157 |
| LF_score_ | Estim. | 0.028^*^ | 0.022^*^ | 0.014^*^ | 0.010^*^ | 0.009^*^ | 0.022^*^ |
|  | SE | 0.002 | 0.002 | 0.001 | 0.001 | 0.002 | 0.001 |
| HF_score_ | Estim. | -0.023^*^ | -0.021^*^ | -0.015^*^ | -0.012^*^ | -0.012^*^ | -0.021^*^ |
|  | SE | 0.002 | 0.001 | 0.001 | 0.001 | 0.002 | 0.001 |
| AVGHR | Estim. | -0.019^*^ | -0.008^*^ | -0.007^*^ | -0.005^*^ | -0.003^*^ | -0.017^*^ |
|  | SE | 0.002 | 0.002 | 0.001 | 0.002 | 0.002 | 0.001 |
| Age | Estim. | 0.028^*^ | 0.030^*^ | 0.024^*^ | 0.024^*^ | 0.022^*^ | 0.024^*^ |
|  | SE | 0.003 | 0.002 | 0.001 | 0.002 | 0.002 | 0.002 |
| Mean speed | Estim. | 0.109^*^ | 0.117^*^ | 0.109^*^ | 0.107^*^ | 0.108^*^ | 0.100^*^ |
|  | SE | 0.001 | 0.001 | 0.001 | 0.001 | 0.001 | 0.001 |

Mean (SE with 2000 samples bootstrapping), ^*^*p*<0.05
